# Continuous Intravenous Sub-Dissociative Dose Ketamine Infusion for Managing Pain in the Emergency Department

**DOI:** 10.5811/westjem.2017.12.36174

**Published:** 2018-03-08

**Authors:** Sergey Motov, Jefferson Drapkin, Antonios Likourezos, Tyler Beals, Ralph Monfort, Christian Fromm, John Marshall

**Affiliations:** Maimonides Medical Center, Department of Emergency Medicine, Brooklyn, New York

## Abstract

**Introduction:**

Our objective was to describe dosing, duration, and pre- and post-infusion analgesic administration of continuous intravenous sub-dissociative dose ketamine (SDK) infusion for managing a variety of painful conditions in the emergency department (ED).

**Methods:**

We conducted a retrospective chart review of patients aged 18 and older presenting to the ED with acute and chronic painful conditions who received continuous SDK infusion in the ED for a period over six years (2010–2016). Primary data analyses included dosing and duration of infusion, rates of pre- and post-infusion analgesic administration, and final diagnoses. Secondary data included pre- and post-infusion pain scores and rates of side effects.

**Results:**

A total of 104 patients were enrolled in the study. Average dosing of SDK infusion was 11.26 mg/hr, and the mean duration of infusion was 135.87 minutes. There was a 38% increase in patients not requiring post-infusion analgesia. The average decrease in pain score was 5.04. There were 12 reported adverse effects, with nausea being the most prevalent.

**Conclusion:**

Continuous intravenous SDK infusion has a role in controlling pain of various etiologies in the ED with a potential to reduce the need for co-analgesics or rescue analgesic administration. There is a need for more robust, prospective, randomized trials that will further evaluate the analgesic efficacy and safety of this modality across a wide range of pain syndromes and different age groups in the ED.

## INTRODUCTION

### Background

Ketamine is a non-competitive N-methyl-D-aspartate (NMDA)/glutamate receptor complex antagonist that reduces pain by diminishing central sensitization, hyperalgesia, and “wind-up” phenomenon at the level of the spinal cord (dorsal ganglion) and central nervous system.^1^ Ketamine administration in a sub-dissociative dosing range (0.1–0.3 mg/kg) leads to anti-hyperalgesia, anti-allodynia, and anti-tolerance, making it useful in managing a variety of acute and chronic painful conditions without adversely affecting hemodynamics and cognition.^1^–^3^ In the emergency department (ED), sub-dissociative dose ketamine (SDK) was found to be effective for patients with acute traumatic and non-traumatic pain, chronic and cancer pain, opioid-tolerant pain and opioid-induced hyperalgesia states.^4^

### Importance

A large body of evidence supports the use of SDK analgesia administered either as an adjunct to opioids or as a single agent in the ED and in the prehospital setting that leads to significant pain relief and opioid sparing.^3^–^10^ Several strategies of SDK administration in the ED exist that include intravenous (IV) push dose (over 2–5 minutes), which is associated with highest rates of minor but bothersome psychoperceptual side effects (feeling of unreality), or short infusion (over 15 minutes) that results in significant decrease of such side effects with preserved analgesic efficacy.^11^,^12^ However, there is virtually no data evaluating the role of continuous SDK infusion in the ED. A study by Ahern et al. that evaluated analgesic efficacy of continuous ketamine infusion lasting for one hour in ED patients with acute pain demonstrated clinically significant pain reduction (change in numerical rating scale [NRS] >3) at 60 and 120 minutes post-administration in 65% and 68% patients, respectively.^5^

### Goals of This Investigation

The goal of our investigation was to evaluate feasibility (dosing, duration and co-analgesics administration), analgesic efficacy, and side-effects profile of continuous SDK infusion in order to manage various acute and chronic painful conditions in the ED. We hypothesized that this analgesic modality can be used in the ED and its administration might result in adequate pain relief with minimal risk for adverse effects.

## METHODS

### Study Design and Setting

We retrospectively reviewed medical charts of patients who were admitted to our ED and received continuous SDK infusion over a six-year period (2010–2016). The study was conducted at a 711-bed urban community teaching hospital with an annual ED census of greater than 120,000 visits. In our ED, ketamine infusions are prepared by the ED pharmacists and administered by nursing staff via infusion pump. The continuous, weight-based SDK infusion order sets are built into our electronic medical record (EMR) system (Allscripts™) with a starting dose of 0.15mg/kg/hr that is titrated upward every 30 minutes by 2.5–5mg as determined by the treating physician. We defined pre-infusion analgesia as an administration of any analgesics deemed necessary by a treating ED clinician prior to initiation of continuous ketamine infusion. Post-infusion analgesia was defined as an administration of opioid and/or non-opioid analgesic from the end of the infusion until patient’s final disposition from the ED. All data with respect to doses and types of analgesics administered to each patient enrolled in the study (ketamine bolus dose, opioid and non-opioid) was aggregated and described as a percentage of total amount of analgesics given pre- and post-infusion. All patients underwent continuous cardiac monitoring and pulse oximetry. This study was approved by the hospital’s institutional review board.

**Population Health Research Capsule**

What do we already know about this issue?

SDK analgesia administered either as an adjunct to opioids or as a single agent in the ED and in prehospital settings leads to significant pain relief and opioid sparing.

What was the research question?

We sought to evaluate the feasibility, analgesic efficacy and side-effects profile of continuous intravenous SDK infusion in the ED.

What was the major finding of the study?

The mean SDK dose was 11 mg/hr with mean duration of 136 minutes, and mean pain scores (NRS) were 7.6 and 2.6 pre-/post-infusion.

How does this improve population health?

Continuous SDK infusion can be used in the ED for a wide range of acute and chronic painful conditions and age groups either as an adjunct to opioid and non-opioid analgesics or as a single agent.

### Selection of Participants

Patients 18 and older presenting to the ED with a variety of acute and chronic painful conditions and receiving a continuous SDK infusion in the ED were eligible for the study. We excluded patients if they received a ketamine infusion for the purpose of sedation, end-of-life care, or received only a bolus dose of ketamine.

### Methods and Measurements

We performed data collection by querying the ED EMR database. Extracted data included age, sex, chief complaint, final diagnoses, pre- and post-infusion NRS pain score, duration of infusion, analgesics given before and after infusion, and adverse effects.

### Outcomes

The primary outcomes of the study were the following: 1) mean dose and duration of the continuous ketamine infusion, 2) percentage of patients receiving analgesics before and after ketamine infusion, and 3) percentage of patients receiving SDK bolus dose prior to continuous ketamine infusion. Secondary outcomes consisted of 1) change in pain score before and after infusion administration via standard 11-point NRS score, and 2) overall rates of adverse effects.

### Data Analysis

The data analyses consisted primarily of descriptive statistics. We described baseline characteristics of patients in each treatment group in terms of mean ± standard deviation for continuous variables and frequency (percent) for categorical variables. A student’s t-test was used to compare simple group differences in terms of means (e.g., age), while we used the chi-square test to look at differences in terms of percent rates (e.g., sex). We carried out all statistical analyses using SPSS® version 24.

## RESULTS

**Figure 1:**
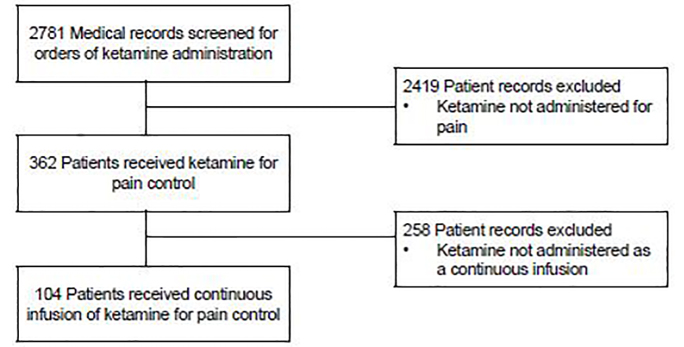
Study flow diagram for selection of patients who received continuous ketamine infusion for pain control.

We reviewed 2,781 medical records containing orders for ketamine dosing, which occurred between January 2010 and December 2016. Of those, we excluded 2,677 patient records due to ketamine use other than a continuous infusion for analgesia. The remaining 104 subjects receiving a continuous SDK infusion for pain control were enrolled into our study (Figure 1).

The mean age was 49.5 years old respectively, with 43% male patients. Mean baseline NRS pain score was 7.63. Most patients presented with chief complaints related to musculoskeletal pain (40.4%) and abdominal pain (36.6%), which roughly correlated with final diagnoses (Tables 1 and 2).

### Main Outcomes

**Figure 2:**
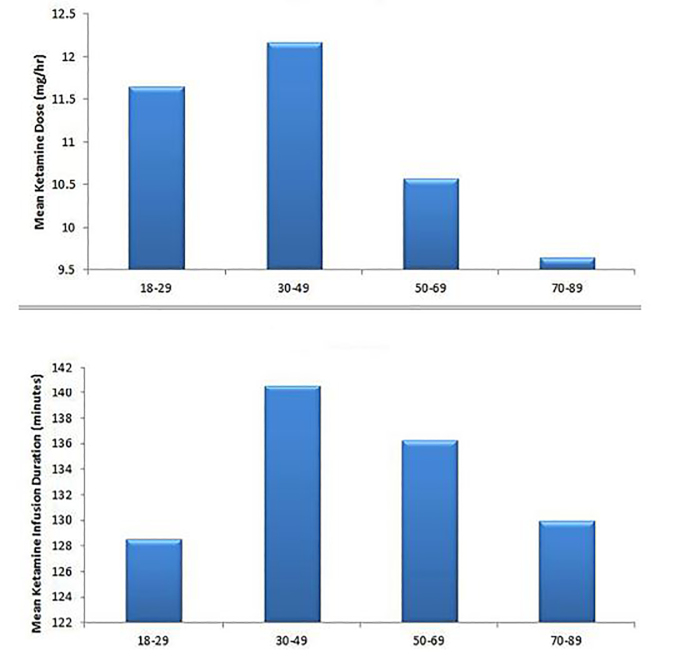
Mean ketamine infusion dose and duration for different age groups.

The overall mean dose for SDK infusion was 11.26 mg/hr (6.0–22.50 mg/hr) with an overall mean duration of treatment of 135.87 minutes (20–480 minutes). When we compared dose and duration of infusion in four different age groups (18–29, 30–49, 50–69, 70–89), we found that patients in the 30–49 age group received the highest mean dose of continuous SDK infusion of 12.17 mg/hr, as well as the longest mean duration of infusion of 140.6 minutes. There was a trend towards lower dosing and shorter duration of infusion in patients 50 years of age and older (Figure 2).

Table 3 shows the percentages of patients receiving analgesia before and after continuous ketamine infusion.

Non-opioid analgesics had the highest rates of pre-infusion administration (38.4% of patients). In addition, 11.5% of patients received no analgesics prior to infusion. Post-infusion, opioids constituted the largest class of analgesics administered (23.1% of patients) with 50% of patients receiving no additional analgesics. Furthermore, 59.6% of patients received an SDK bolus before the continuous infusion, and 11.5% of patients received the continuous SDK infusion alone.

**Figure 3:**
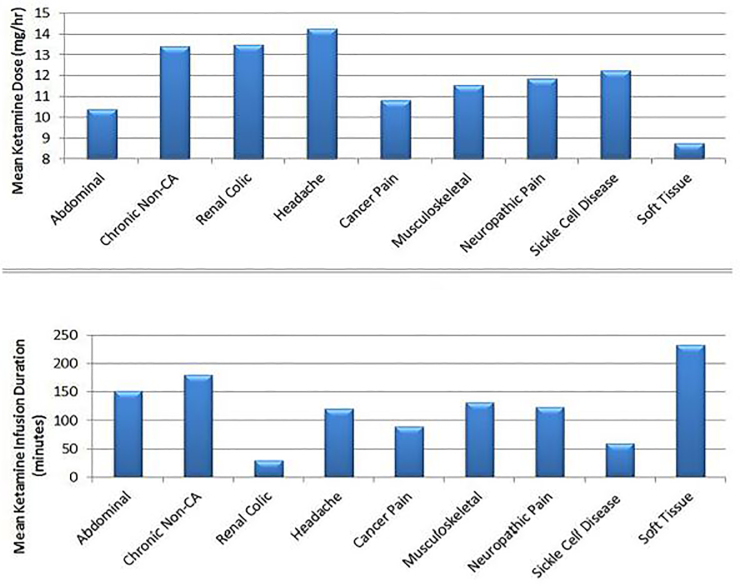
Mean ketamine infusion dose and duration for final diagnosis groups.

Furthermore, upon comparing the dose and duration of continuous SDK infusion across the nine final diagnoses, we found that patients with a clinical diagnoses of headache, renal colic, and chronic non-cancer pain received the highest doses of continuous ketamine infusion. Patients with a clinical diagnoses of soft tissue, chronic non-cancer, and abdominal pain on average had the longest duration of ketamine infusion (Figure 3).

**Figure 4:**
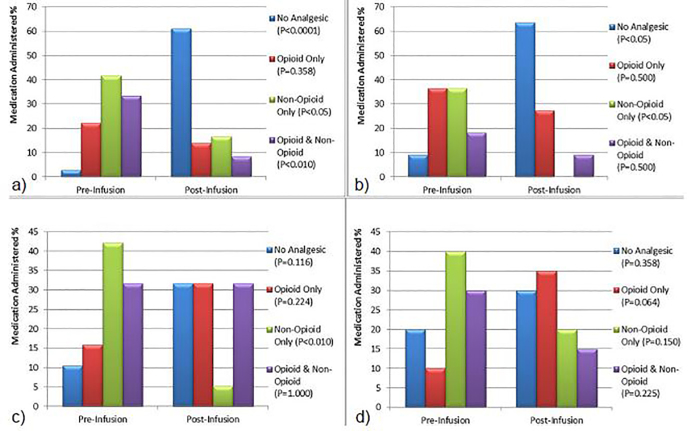
Analgesics administered pre- and post-ketamine infusion for most common final diagnoses; a) abdominal pain, b) cancer pain, c) musculoskeletal pain, d) neuropathic pain.

Administration of analgesics before and after continuous SDK infusion varied greatly between the four most prevalent clinical diagnoses groups, most notably in patients with abdominal and cancer pain. Patients with abdominal pain demonstrated the largest difference in not receiving any analgesic from 2.8% pre-infusion to 61.1% post-infusion (p<0.0001). Patients with cancer pain also showed a significant difference of not receiving any analgesic from 9.1% pre-infusion to 63.6% post-infusion (p<0.05). These findings were noted despite the fact that abdominal-pain and cancer-pain patients received relatively lower doses of ketamine. While no definitive conclusion can be drawn from this observation, it may suggest a higher rate of analgesic efficacy in the two patient populations. Furthermore, patients with abdominal and cancer pain had reduced requirements for non-opioid analgesics post-continuous ketamine infusion (p<0.05) **(**Figure 4). Patients with musculoskeletal pain exhibited a significant decrease in non-opioid analgesic administration post infusion (p<0.010) while patients with neuropathic pain showed an increase in opioid-only analgesic administration (p=0.064) (Figure 4).

### Secondary Outcomes

Complete NRS data was not available for all 104 patients. For 53.8% of patients with available pain scores, the mean NRS pain scores were 7.63 (±2.3) pre-infusion and 2.65 (±3.3) post-infusion, resulting in an average pain score decrease of 5.04 (95% confidence interval [CI] [4.07–6.00]; p<0.0001). Furthermore, for patients with one of the top three diagnoses of abdominal, musculoskeletal, and neuropathic pain with available pre- and post-pain scores, the mean decrease in pain score was 4.95 (95% CI [3.36–6.53]; p<0.0001) for abdominal pain, 4.78 (95% CI [1.28–8.28]; p<0.05) for musculoskeletal pain, and 3.69 (95% CI [1.45–5.93]; p<0.005) for neuropathic pain. Five adverse effects were documented in a total of 12 patients: nausea (5.8%), headache (1.9%), dizziness (1.9%), rash (1.0%), and confusion (1.0%). Ninety-two (88.4%) patients had no documentation of any adverse effects. Two (1.9%) patients presenting with abdominal and renal colic pain required discontinuation of SDK infusion due to severe nausea. Thirty-six (34.6%) patients were admitted to the hospital for further pain control after infusion was completed, which included 13 (12.5%) with abdominal pain, six (5.8%) with cancer pain, five (4.8%) with musculoskeletal pain, five (4.8%) with neuropathic pain, three (2.9%) with sickle cell pain, three (2.9%) with chronic pain, and one (0.9%) with flank pain.

## LIMITATIONS

The retrospective nature of our study, relatively small sample size, and lack of documented pain scores in 46.2% of patients were the major limitations. As a result, we could not fully evaluate and compare the analgesic efficacy of continuous SDK infusion between different age groups and between different pain syndromes; thus, we could not assert any recommendation with respect to overall pain relief. Furthermore, due to the fact that only 53.8% of patients had documented pre- and post-infusion pain scores and only 59.6% of patients received a ketamine bolus prior to the infusion we could not accurately and reliably compare the difference in improvement of pain scores between patients receiving a bolus dose followed by infusion to infusion only. Additionally, since the primary outcome of the study was the dosing regimen for continuous ketamine infusion, dosages for analgesics given to patients before and after infusion were not abstracted. Lastly, due to the retrospective nature of the study we cannot make any statements regarding the safety of continuous SDK infusion in our ED. Future prospective studies are needed to evaluate the safety and efficacy of SDK infusion in the ED.

## DISCUSSION

SDK administration in the form of IV push or short infusion is becoming increasingly popular as a viable adjunct to or even a substitute for opioid analgesics in managing a variety of acute and chronic painful conditions in the ED.^3^–^12^ To date, however, there is a paucity of data that supports the use of continuous SDK infusion (longer than one hour) in the ED. Ahern et al. prospectively administered 15 mg of IV SDK immediately followed by a continuous infusion of 20 mg/hr for one hour to 38 ED patients with acute pain. At the one-hour mark, 25 and 26 patients had significant pain relief (NRS reduction greater than 3) at 60 and 120 minutes, respectively.^5^

A growing body of literature advocates for use of continuous SDK infusion either as an adjunct to opioids or as a single agent for pediatric and adult patients with predominantly chronic painful conditions. A recent cohort study that included 230 hospitalized patients receiving continuous ketamine infusion demonstrated a 34% decrease in pain score after one day of treatment. In addition 58% of patients achieved equal or greater than 20% overall reduction in pain scores without psychotomimetic side effects requiring therapy. Furthermore, patients with cancer pain and patients with pancreatitis and Crohn’s disease had greater reductions in pain scores.^13^

A retrospective chart review of five pediatric patients with sickle cell disease and acute vaso-occlusive crisis who received continuous ketamine infusion with a dosing range of 0.06 mg/kg/hr to 0.1 mg/kg/hr and duration of treatment from 19 to 90 hours, demonstrated a clinically significant pain reduction in two children and reduction in opioid consumption in one child. Two patients experienced side effects (mainly dysphoria) that resulted in treatment termination in one patient.^14^ A case report of continuous ketamine infusion in adult patients with acute vaso-occlusive painful crisis administered for seven days resulted in 65% pain relief at the end of the treatment course without any psychoperceptual side effects.^15^ Another case report of a patient with post-operative phantom pain and allodynia who was started on ultra-low dose (1.5–5 mg/hr) of continuous ketamine infusion for three days, demonstrated a 60% pain decrease during the initial hours of administration without any psychomimetic side effects.^16^

Our retrospective chart review demonstrates that continuous SDK infusion has the potential to be used in the ED across a wide range of acute and chronic painful conditions and age groups either as an adjunct to opioid and non-opioid analgesics or as a single agent. The fact that continuous SDK infusion alleviated the need for additional post-infusion analgesia in 60% and 55% of patients with abdominal pain and cancer pain is very encouraging even though we could not fully evaluate the analgesic efficacy of this analgesic modality due to the retrospective nature of this study. In addition, our chart review showed that patients with neuropathic pain and chronic non-cancer pain required higher rates of post-infusion opioid rescue analgesia and a longer duration of ketamine infusion, thus demonstrating that management of such painful conditions in the ED can be very challenging.

Lastly, our data showed that continuous SDK infusion can be employed for geriatric patients with a broad range of painful syndromes in the ED, thus adding an additional analgesic modality when opioids and non-steroidal anti-inflammatory drugs are contraindicated.

One of the possible barriers to use of continuous SDK infusion are potential administrative concerns regarding an off-label use of an anesthetic agent such as ketamine for managing pain in the ED and on the hospital wards. Departmental and interdisciplinary protocols with clearly specified, patient eligibility criteria as well as indications for and contraindications to SDK infusion should be in place before widespread use of this analgesic modality is considered for implementation.

## CONCLUSION

Continuous intravenous SDK infusion does have a role in controlling pain of various etiologies in the ED with the potential added benefit of decreased need for additional analgesia. There is a need for more robust, prospective, randomized trials that will further evaluate the analgesic efficacy and safety of this analgesic modality across a wide range of pain syndromes and different age groups.

## Figures and Tables

**Table 1:** Characteristics of patients receiving continuous ketamine infusion for pain in the emergency department.

Patients (104)	N (%)

Characteristic	
Sex	
Male	45 (43.3)
Female	59 (56.7)
Age, mean (median)	49.51 (49)
Chief complaint	
Musculoskeletal pain	42 (40.4)
Abdominal pain	38 (36.6)
Flank pain	4 (3.8)
Sickle cell disease	4 (3.8)
Other (chronic, non-cancerous, neuropathic, soft tissue pain)	16 (15.4)

**Table 2:** Patient clinical diagnosis.

Final diagnosis	N (%)
Abdominal pain (n=36)	
Non specific	21 (20.2)
Pancreatitis	6 (5.8)
Cyclic vomiting	3 (2.9)
Bowel obstruction	2 (1.9)
Gastroparesis	2 (1.9)
Gastritis	1 (1)
Cholecystitis	1 (1)
Musculoskeletal pain (n=19)	
Back (generalized/spasm)	10 (9.6)
Extremity (fracture/dislocation)	6 (5.8)
Ribs (trauma/fracture)	3 (2.9)
Neuropathic pain	20 (19.2)
Cancer pain	11 (10.6)
Chronic non-cancer pain	5 (4.8)
Sickle cell disease	4 (3.8)
Renal colic	3 (2.9)
Soft tissue pain	3 (2.9)
Headache	2 (1.9)
Other	1 (1)

**Table 3:** Analgesics administration pre- and post-ketamine infusion.

Type of Analgesic Pre-Infusion	N (%)
Opioid only	18 (17.3)
Non-opioid only	41 (38.4)
Opioid and Non-opioid	33 (31.7)
No analgesics administered	12 (11.5)
	Type of analgesic
Post-Infusion	N (%)
Opioid only	24 (23.1)
Non-opioid only	13 (12.5)
Opioid and non-opioid	15 (14.4)
No analgesics administered	52 (50)
	Ketamine Bolus
Pre-Infusion	N (%)
Ketamine bolus	
Administered	62 (59.6)
Not administered	42 (40.4)
Ketamine continuous infusion with no other analgesics	12 (11.5)
